# Case Report: Medical Management of Prolonged Gestation of a Mummified Fetus in a Bitch

**DOI:** 10.3389/fvets.2022.888807

**Published:** 2022-06-02

**Authors:** Annemarie Spruijt, Lucinda van Stee, Karin Wolthers, Jeffrey de Gier

**Affiliations:** Department of Clinical Sciences, Faculty of Veterinary Medicine, Utrecht University, Utrecht, Netherlands

**Keywords:** aglépristone, canine, fetal death, mummification, parturition induction, PGF2α

## Abstract

A 3-year-old female rough coated collie was presented at day 69 (D69) after the first mating. She was mated on 2 consecutive days based on ovulation timing by the referring veterinarian. At day 30 post breeding, a single, live embryo was seen on ultrasound by this veterinarian. On D69, the bitch was presented to us because she lacked signs of impending parturition such as vulvar discharge or nest building behavior. On general examination, the bitch appeared clinically healthy and no prodromi were present. On abdominal palpation a small, firm structure and a slightly enlarged uterus were detected. There was no vulvar discharge. Using vaginoscopy we could not see any signs of cervical dilatation. Additionally, ultrasonography revealed the presence of a collapsed fetus in the uterus with a moderate amount of echogenic fluid surrounding it and the plasma progesterone concentration was 2.6 ng/ml. A parturition induction protocol was initiated: a progesterone receptor antagonist was administered, followed by PGF2α to induce cervical relaxation and uterine contractions. The fetus was expelled 3 days later, without noticeable damage to the reproductive tract of the dam. The bitch subsequently delivered two more litters without complications. To our knowledge this is the first clinical report that demonstrates a successful *non-surgical* treatment to expel a mummified fetus after *prolonged* gestation. The pharmacological treatment did not affect the future fertility of the breeding dog, which is an important outcome for breeders.

## Introduction

This case describes the pharmacological treatment of prolonged gestation of a 3-year-old primiparous rough coated collie breeding bitch (15 kg) in which the process of parturition of a mummified fetus did not start spontaneously. The duration of gestation in the bitch from the day of mating at 2 days post ovulation varies between 58 and 65 days with a mean of 61.4 ± 1.5 days (1).

Corpora lutea appear to be the only source of progesterone during pregnancy (2) and, therefore, luteolysis, resulting in a decline in progesterone concentration, is necessary to initiate parturition. Factors involved in the initiation of parturition seem to be cortisol, oxytocin and relaxin which may trigger prostaglandin synthesis which results in luteolysis (3, 4). However, the prepartum luteolytic cascade in dogs is still not known in detail. According to Kowalewski et al. (5, 6), there is a role for the fetal trophoblast cells in this process because they seem to be the major source of prepartum placental prostaglandins. Additionally, under the influence of PGF2α, myometrial activity gradually increases, which is essential for the onset of whelping (7). Depending on the cause, the stage of gestation and the immunological interaction between mother and fetus, fetal death can result in mummification, maceration, or in expulsion (8). Mummification can occur as a result of autolytic changes in the fetus, absorption of placenta and fetal fluids and involution of the maternal placenta (9). Fetal mummification is to be differentiated from fetal maceration, in which the fetus putrefies in the uterine cavity in the presence of bacteria and oxygen originating from the open cervix. If maceration occurs after bone formation, autolysis would continue until fetal soft tissues become autolyzed and only bones remain in the uterus (10). It is suggested that the process of mummification takes several weeks, depending on the age of the fetus at time of dead. It usually occurs in the last stage of gestation after ossification of the bones (11). Although fetal mummification has been described in the bitch, the actual incidence is unknown, but presumed to be very low (9, 11). Complications associated with the presence of a dead fetus in a dog include uterine perforation, endometritis, preperforative peritonitis, and even ectopic pregnancy (9, 10). Based on the fact that we know that bacterial invasion can develop rapidly in the canine uterus and lead to toxemia and septicemia, a dead fetus may have fatal consequences (9, 12, 13). Previously published case reports on the treatment of canine mummification were mostly based on surgical interventions like cesarean section or unilateral or complete ovariohysterectomy (11, 14). However, in cases of prolonged pregnancy in bitches carrying only one or more mummified fetuses, successful medical treatment would be preferable particularly in cases where there is intent to retain reproductive capacity. Protocols for induction of parturition might be appropriate for those patients in which plasma progesterone concentrations has not yet decreased to basal values, for example including the use of a progesterone antagonist (3, 15) either or not combined with prostaglandins (16) and/or oxytocin (17–19). To our knowledge there is no study that describes a successful *non-surgical* treatment to expel a mummified fetus after *prolonged* gestation.

## Case Description

**Figure 1:**
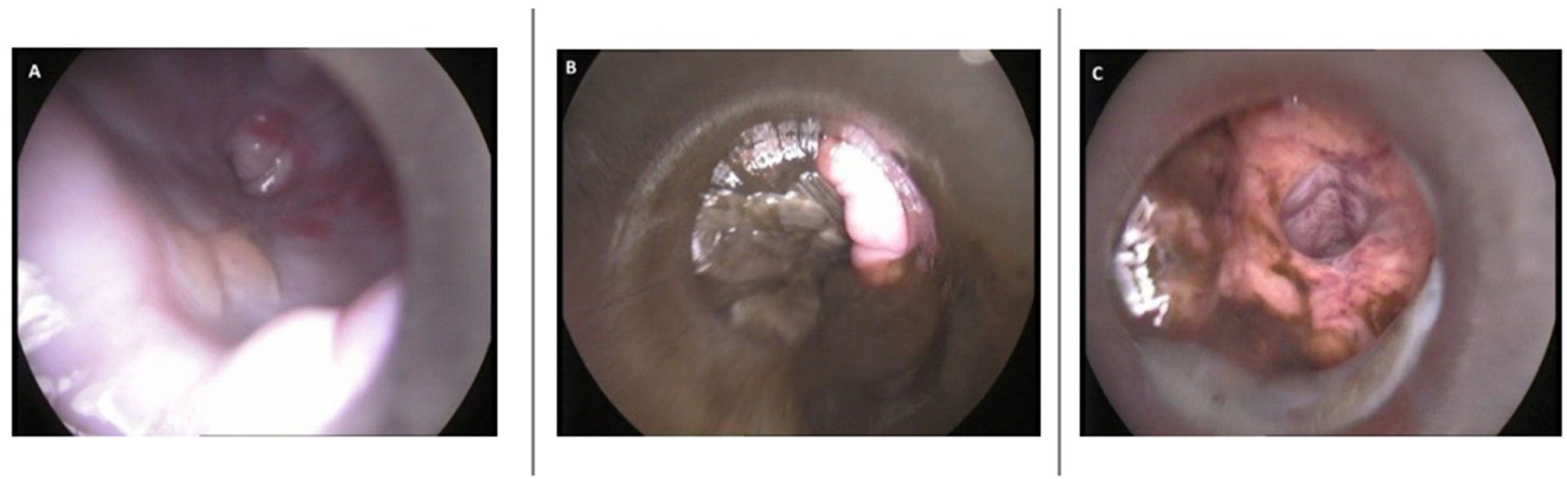
Images of vaginoscopy. **(A)** Aspect of cranial vaginal mucosa showing red and pale colored patchwork. A small amount of clear yellow mucus can be seen ventrally (D69). **(B)** Fetal membranes and dark green mucous discharge in vagina (D71). **(C)** A wide uterine lumen with green mucous discharge confirming open cervix (D72).

**Figure 2:**
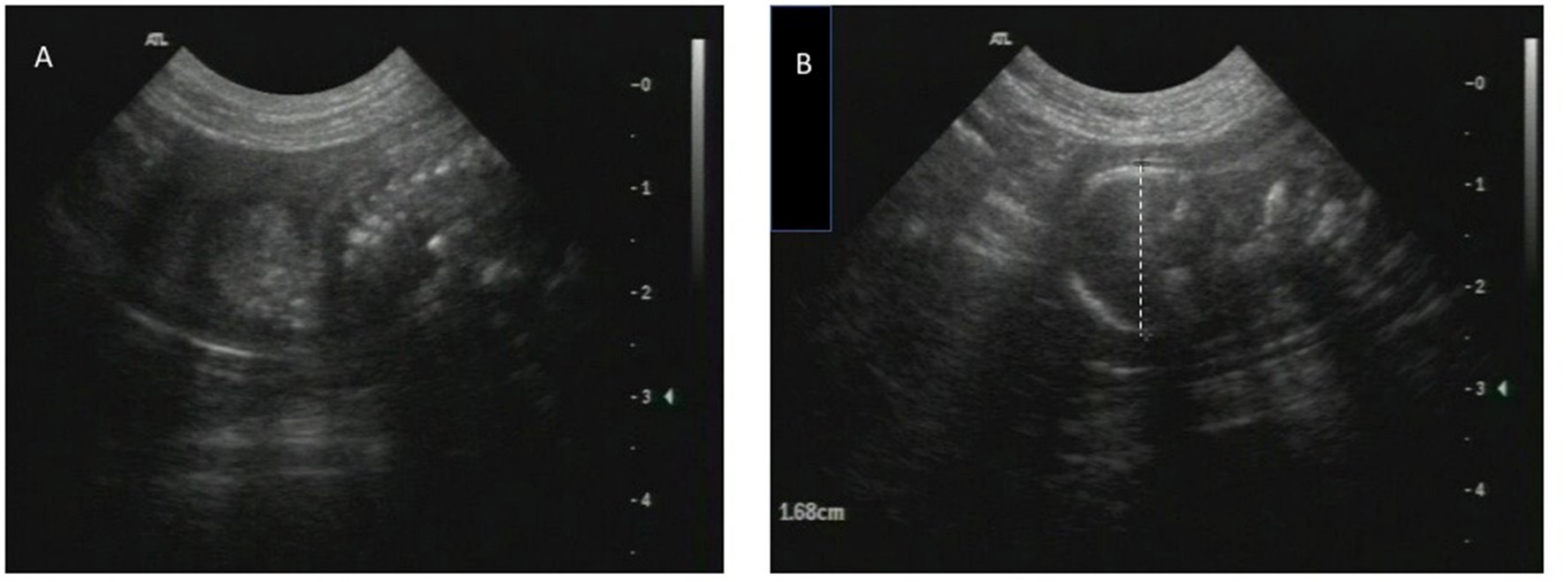
Images of ultrasound. **(A,B)** A collapsed fetus in the left uterine horn with BPD of 16.8 mm surrounded by echogenic fluid (D69).

A 3-year-old primiparous rough coated collie bitch (15 kg) was presented to the University Clinic for Companion Animals (UCCA) at Utrecht University on D69 after the first mating. Ovulation timing was performed by the referring veterinarian. At day 11 after the start of pro-estrus, progesterone was 6.2 ng/ml and the bitch was mated on two consecutive days (D12 and D13). On day 30 after the first mating, the referring veterinarian palpated one embryonic vesicle. Ultrasonographic evaluation of the uterus showed one, viable embryo. On D61 of gestation the owner presented the bitch to their veterinarian as no signs of imminent parturition were present yet. An abdominal radiograph revealed the Spalding sign, the fetus had demised. The veterinarian prescribed Amoxicillin plus Clavulanic acid (Synulox 250 mg orally) at a dosage of 16,7 mg/kg twice a day to prevent infection and referred the bitch to the UCCA. At the day of presentation (D69), the bitch was clinically healthy, there was no vulvar discharge and no prodromi were present. On abdominal palpation a small, firm structure and a slightly enlarged uterus were detected. Vaginoscopy, performed with a pediatric proctoscope (cat. no. 8834.08, R. Wolf, Germany) connected to a light source (cat. no. 4220 LP, R. Wolf, Germany), showed unswollen vaginal mucosa with patchwork-like pattern of red and pale areas. The cranial part of the dorsal median fold obscured the view on the cervix, but no indications of cervical dilation were found. A small amount of clear yellow mucus was seen ventrally in the cranial portion of the vagina (Figure 1A). No fetal membranes, lesions, or other pathologies were seen in vagina or vestibulum. The plasma progesterone concentration was 2.6 ng/ml [^125^I RIA, validated for fertility breeding management (1)]. Ultrasonographic evaluation of the uterus, performed by an ECVDI diplomate, revealed a collapsed fetus in the left uterine horn (Figures 2A,B) with biparietal diameter (BPD) of 16.8 mm surrounded by echogenic fluid. Total uterine thickness of the left uterine horn was 9 mm, with a uterine wall thickness of 2.6 mm. At this stage, the diagnosis of a mummified fetus was made. In order to assess possible systemic effects due to the mummified fetus, a complete blood cell count was performed, and plasma creatinine was determined, which were all within normal limits (Table 1).

**Table 1:** Results of hematological, biochemical, and endocrine analyses on D69.

**Parameter**	**Value**	**Reference value**
Hematocrit	0.40 L/L	0.42–0.61L/L
Leucocytes	6.0 ×10^9^/L	4.5–14.6 ×10^9^/L
Polym. Nucl.	3.9 ×10^9^/L	2.9–11.0 ×10^9^/L
Rods	-	0.0–0.3 ×10^9^/L
Juveniles	-	0.0 ×10^9^/L
Lymphocytes	1.5 ×10^9^/L	0.8–4.7 ×10^9^/L
Monocytes	0.3 ×10^9^/L	0.0–0.9 ×10^9^/L
Eosinophils	0.3 ×10^9^/L	0.0–1.6 ×10^9^/L
Basophils	-	0.0–0.1 ×10^9^/L
Normoblasts	-	
Creatinine	96 μmol/L	50–129 μmol/L
P4	2.6 ng/ml	

**Figure 3:**
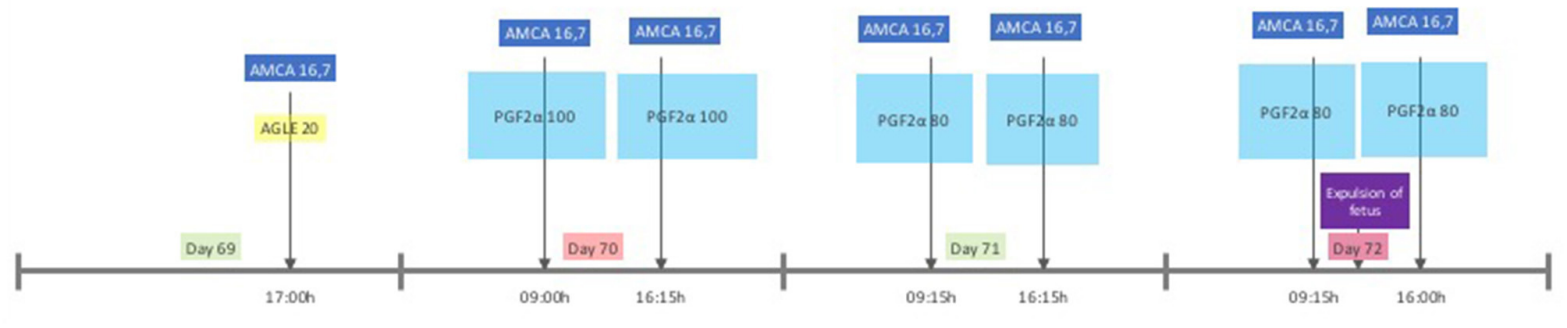
Protocol, timeline, and dose of medical treatment. AGLE dose in mg/kg BW (SC); PGF2a dose in μg/kg BW (IM); AMCA 16.7 mg/kg BW (OR).

Because medical complications were absent at the moment of presentation and the owners wished to maintain her breeding ability, medical management was prioritized above surgery. Therefore, a parturition induction protocol (Figure 3) was created using the progesterone antagonist aglépristone (Alizin, Virbac, Barneveld, The Netherlands) to induce cervical dilation and PGF2α dinoprost (Dinolytic, Zoetis, Capelle aan de IJssel, The Netherlands) to induce myometrial contractions in order to expel the uterine content. Treatment with Amoxicillin plus Clavulanic acid (Synulox 250 mg twice a day PO) was continued to reduce the risk of development of post-partum endometritis. The bitch was hospitalized and monitored closely throughout the treatment because a dead fetus in the uterus can be a serious risk for the bitch as bacterial invasion can rapidly develop and may have fatal consequences (9). Before every PGF2α administration, a general physical examination was performed, supplemented with abdominal palpation, examination of the vulva and peri vulvar area for discharge. On D69, the treatment was initiated with a single dose of aglépristone, at 20 mg/kg BW SC, divided in portions of no more than 5 ml per injection site. Fifteen hours after aglépristone administration, PGF2α was administered at an initial dose of 100 μg/kg (IM) and repeated twice daily. A summary of the treatment from D69 to D72 can be found in Figure 3. Before the second administration of PGF2α (D70), moderate vaginal discharge of dark green mucus was observed which was homogenous and had a slightly malodorous smell. On D71 the ultrasound confirmed that the mummy moved to the pelvic entrance and vaginoscopy showed fetal membranes and dark green mucous discharge in the vagina (Figure 1B). The discharge became less viscous and less abundant up to D72 of gestation. While in the morning of D72 the mummy was still palpable in the pelvic entrance via abdominal palpation, later that day vaginoscopy and hysteroscopy confirmed an open cervix, wide uterine lumen with dark green mucus and remnants of fetal tissue (Figure 1C). The fetus was not found, and we concluded that the mummy was expelled after the fifth PGF2α administration and suggested that the bitch ate it. Observed physical reaction to the administration of aglépristone was discomfort during injection. Observed side effects of the administration of PGF2α were diarrhea, vomiting, panting, digging behavior and restlessness. Vomiting and diarrhea ceased after the dosage was decreased by 20% to 80 μg/kg BW. All observed side effects of PGF2α started within a few minutes after administration and lasted for approximately 1 h.

After the fetus was expelled, the dog was sent home with the owner and additionally treated with oxytocin (Oxytocin, Eurovet Animal Health, Bladel, The Netherlands) 5 IU four times a day SC for 2 days and antibiotics for another 7 days to reduce the risk of development of post-partum endometritis. The owner was instructed to measure the rectal temperature twice daily until the revisit 7 days later. Body temperature remained within reference limits. No side effects of the oxytocin administration were noted. On the day of the revisit, a general physical examination revealed no abnormalities. On abdominal palpation, the uterus felt firm and enlarged with a diameter of approximately 25 mm of the corpus uteri. No fluctuation was present. Gynecological examination showed small amounts of white to pink slightly viscous discharge on the vulvar cleft but no further abnormalities. The mammary glands were not enlarged nor productive. Consequently, the treatment was stopped. The bitch was successfully bred at the subsequent estrus 6 months later, resulting in a litter of 3 pups and thereafter, she raised another litter without any complications.

## Discussion

This case gives input for the discussion on the onset of parturition in the dog from a clinical perspective and provides a protocol for parturition induction of a dead fetus in a prolonged pregnancy. Data on fetal hormone secretion that initiate this luteolytic process in dogs are lacking, but according to Kowalewski et al. (5) there seems to be a role for the fetal trophoblast cells as the major source of prepartum secretion of placental prostaglandins. There is also evidence that other intraluteal processes, such as invasion of macrophages, massive expression of apoptotic enzymes and significantly decreased expression of the steroidogenic regulatory proteins, are involved in completion of prepartum luteolysis when the influence of progesterone is declining (20). In our case, the gynecological examination, ultrasound findings and non-basal plasma progesterone concentration at D69 of pregnancy indicated that luteolysis was not complete and suggested that the onset of parturition was not initiated. A possible explanation could be that the fetal trophoblast cells were not active anymore or that the lack of feto-maternal interaction prohibited the luteolytic cascade that leads to parturition. While we do not know exactly when the fetus had died, we expect this to be *after* D41 (range D39-D42) of gestation based on Concannon (21) who states that the first evidence of mineralization of the fetal skull start on D45 (range D43–D46) after LH Surge and fetal skull was mineralized at the moment of presentation at our clinic. In addition, according to the formula designed by Lopate (22) to calculate gestational age, the estimated day of gestational age in a medium breed dog with a BPD of 16.8 mm is 46 days after LH surge, which roughly corresponds to D42 from conception. However, this formula is based on living fetuses in a non-complicated pregnancy. In our case is it reasonable that development of the fetus was already arrested and that the BPD was smaller than in “normal” fetuses. This calculation can thus lead to a shorter gestational age than it actually was. The protocol used in the present case, was a modification of a protocol described by Hoffmann et al. (16) for termination of a prolonged gestation with living fetuses. Compared to that, we combined the two initial doses of aglépristone in one administration (20 mg/kg BW) with a different dose of PGF2α (100 μg/kg BW) with almost the same interval (12–16 h after aglépristone). Other authors that studied parturition induction protocols with living fetusus in bitches that were near term (15, 17–19) used a dose of 15 mg/kg BW aglépristone and repeated that after 9 h (15) or the next day (17–19). We choose a higher dose of aglépristone in one administration based on both the slow absorption from the injection site and slow excretion (23) in combination with the practical considerations. The reason we choose oxytocin instead of PGF2α as ecbolic after the expel of the fetus and discharge of the patient from our hospital was because, in our opinion, the usage of PFG2α requires hospitalization because of the small therapeutic range and possible side effects. However, we are aware that this was without scientific evidence that this would have an effect at this moment after parturition, especially in this case, meaning this long after administration of aglépristone.

Efendic et al. (24) described the induction of abortion of a dead conceptus earlier in the pregnancy (D34 post-ovulation) by using a combination of aglépristone (D34 and D35, 10 mg/kg) and cloprostenol daily in diverse concentrations (1–3.5 μg/kg) for 23 days and added misoprostenol (400 μg/kg) intravaginally after 10 days until D52. On D57 a huge amount of dark brown mucosal vaginal discharge was noted. Despite the fact that the medical treatment also resulted in the expulsion of the abnormal uterine content, the case is not comparable to ours because of the important difference in gestational length at the start of treatment. Besides that, that treatment protocol was long and therefore has an increased risk of uterine complications and of bacterial infections and was more costly.

The biggest limitation of this clinical report is the lack of several additional examinations, such as virologic tests. Based on our palpation and hysteroscopy on D3 after treatment we were certain of the expulsion of the mummified fetus. However, when the veterinarian is in doubt, an ultrasound or radiograph should be done to ensure expulsion.

## Conclusion

From this report, it could be concluded that in case of prolonged gestation of a mummified fetus the combination of a progesterone antagonist and PGF2α helps to open the cervix and expel the mummified fetus within 3 days. The protocol did cause some mild and transient side effects but prevented surgery and it did not affect future fertility, as the bitch produced two healthy litters later in her life. However, it is important to hospitalize and monitor the patient during treatment, for complications can arise with a dead fetus in the utero. If complications arise ovariohysterectomy might still be required.

## Data Availability Statement

The original contributions presented in the study are included in the article/supplementary material, further inquiries can be directed to the corresponding author/s.

## Ethics Statement

Ethical review and approval was not required as this is a case report based on one case. Informed consent was obtained from the owner of the animal.

## Author Contributions

AS and LS prepared the first draft. KW and JG helped with manuscript preparation and gave feedback on the draft. All authors read and approved the final manuscript.

## Conflict of Interest

The authors declare that the research was conducted in the absence of any commercial or financial relationships that could be construed as a potential conflict of interest.

## Publisher's Note

All claims expressed in this article are solely those of the authors and do not necessarily represent those of their affiliated organizations, or those of the publisher, the editors and the reviewers. Any product that may be evaluated in this article, or claim that may be made by its manufacturer, is not guaranteed or endorsed by the publisher.

## Abbreviations

AGLE: Aglépristone
AMCA: Amoxicillin plus Clavulanic acid
BW: Bodyweight
P4: Progesterone
PGF2α: Prostaglandin F2 alpha.
